# [Corrigendum] Biological effects of BMP7 on small-cell lung cancer cells and its bone metastasis

**DOI:** 10.3892/ijo.2025.5757

**Published:** 2025-05-21

**Authors:** Weiwei Shen, Hailin Pang, Bo Xin, Lian Duan, Lili Liu, Helong Zhang

Int J Oncol 53: 1354-1362, 2018; DOI: 10.3892/ijo.2018.4469

Subsequently to the publication of the above article, the authors contacted the Editorial Office to explain that, for the cell migration and invasion assay experiments shown in Fig. 3 on p. 1358, the same data had inadvertently been selected to show the results of the 'Migration / SBC-3-rhBMP7' experiment in Fig. 3A and the 'Invasion / SBC-5' experiment in Fig. 3B.

After re-examining their original data, the authors have realized that the data were correctly shown for the 'Migration / SBC-3-rhBMP7' experiment in Fig. 3A. Therefore, the revised version of Fig. 3, now showing the correct data for the 'Invasion / SBC-5' experiment in Fig. 3B, is shown on the next page. The authors are grateful to the Editor of *International Journal of Oncology* for allowing them this opportunity to publish a Corrigendum, and all the authors agree with its publication. Furthermore, the authors apologize to the readership for any inconvenience caused.

## Figures and Tables

**Figure 3:**
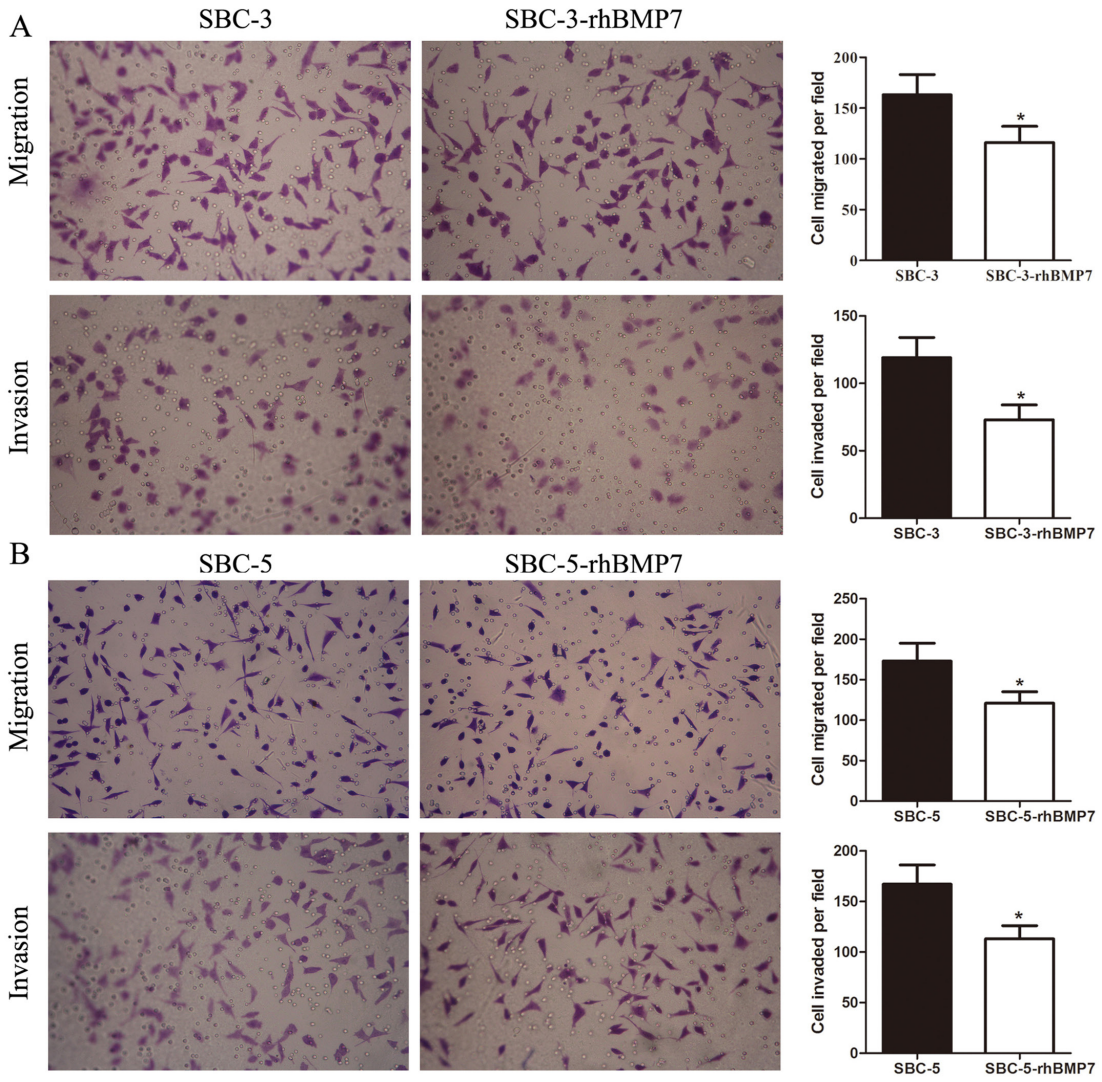
Effect of rhBMP7 on migration and invasion in small-cell lung cancer cells. Representative images, and quantification of the numbers, of migrated and invaded (A) SBC-3 and (B) SBC-5 cells with or without rhBMP7. rhBMP7, recombinant human bone morphogenetic protein 7. ^*^P<0.05.

